# Psychiatric Hospital Bed Numbers and Prison Population Sizes in 26 European Countries: A Critical Reconsideration of the Penrose Hypothesis

**DOI:** 10.1371/journal.pone.0142163

**Published:** 2015-11-03

**Authors:** Victor Blüml, Thomas Waldhör, Nestor D. Kapusta, Benjamin Vyssoki

**Affiliations:** 1 Department of Psychoanalysis and Psychotherapy, Medical University of Vienna, Vienna, Austria; 2 Department of Epidemiology, Center of Public Health, Medical University of Vienna, Vienna, Austria; 3 Department of Psychiatry and Psychotherapy, Clinical Division of Social-Psychiatry, Medical University of Vienna, Vienna, Austria; Medical University of Vienna, AUSTRIA

## Abstract

**Background:**

Recently, there has been a revived interest in the validity of the Penrose hypothesis, which was originally postulated over 75 years ago. It suggests an inverse relationship between the numbers of psychiatric hospital beds and the sizes of prison population. This study aims to investigate the association between psychiatric hospital beds and prison populations in a large sample of 26 European countries between 1993 and 2011.

**Methods:**

The association between prison population sizes and numbers of psychiatric hospital beds was assessed by means of Spearman correlations and modeled by a mixed random coefficient regression model. Socioeconomic variables were considered as covariates. Data were retrieved from Eurostat, the statistical office of the European Union.

**Outcomes:**

Mean Spearman correlation coefficients between psychiatric beds and prison population showed a significant negative association (-0.35; p = <0.01). However, in the mixed regression model including socioeconomic covariates there were no significant fixed parameter estimates. Meanwhile, the covariance estimates for the random coefficients psychiatric beds (σ^2^ = 0.75, p = <0.01) and year (σ^2^ = 0.0007, p = 0.03) yielded significant results.

**Interpretation:**

These findings do not support the general validity of the Penrose hypothesis. Notably, the results of the mixed-model show a significant variation in the magnitude and direction of the association of psychiatric hospital bed numbers and the prison population sizes between countries. In this sense, our results challenge the prevalent opinion that a reduction of psychiatric beds subsequently leads to increasing incarcerations. These findings also work against the potential stigmatization of individuals suffering from mental disorders as criminals, which could be an unintentional byproduct of the Penrose hypothesis.

## Introduction

During the last decades, starting in the 1960s in Europe and in the 1970s in USA, a paradigm shift in treatment of patients with mental disorders has occurred. This process, called deinstitutionalization was aimed at closing asylums, shorten inpatient treatment episodes and—most importantly—reintegrating patients with mental disorders into society [1] [2] [3] [4]. This process was planned to be accompanied by improvements in outpatient treatment facilities, e.g. higher density of psychiatrists and psychotherapists. However, even in high income countries there is a continuous struggle for obtaining sufficient funding for outpatient mental health services [5] [6] [7]

The consequences of this process of deinstitutionalization are manifold and complex. One of the pertinent issues concerns a suggested link between the reduction of psychiatric hospital beds in most countries in the last decades and rising levels of prison populations [8]. The failure to provide adequate psychiatric treatment to individuals suffering from severe mental disorders is suggested to lead to increased levels of criminal behavior and subsequent incarceration of mentally disordered individuals. This theory was first put forward by the British psychiatrist and geneticist Lionel S. Penrose over 75 years ago who found an inverse relationship between the number of and prison populations in 18 European countries [9–11]. This finding came to be known as the *Penrose hypothesis*.

Recently, an article by Mundt et al. [12] has revived interest in the validity of the Penrose hypothesis. Using a longitudinal research design with data from six South American countries since 1990 they found evidence in support of the proposed inverse relationship between psychiatric hospital bed numbers and the sizes of prison population. Their findings remained significant even after controlling for potential confounding economic factors such as economic growth and income inequality. The authors emphasize that the reported association is not just a byproduct of an overall tendency to reduce psychiatric hospital beds and increase prison populations, but an expression of a more direct link between these two variables. Similar findings of a decrease of psychiatric hospital beds and a concurrent increase in prison populations have been reported from Ireland and Norway [13, 14].

However, research findings have not been unanimous and a number of studies did not find evidence in support of the Penrose hypothesis. A worldwide analysis using data from 158 countries showed a positive correlation between psychiatric beds and prison populations in low-and-middle-income countries and found no significant relationship in high-income countries [15]. There is also data from longitudinal studies from the US and several former communist countries casting doubt on the universal validity of the Penrose hypothesis [16, 17]. Political, socioeconomic, and other complex regional differences are hypothesized to play a major role in the postulated association between psychiatric beds and prison populations.

Many of the aforementioned studies suffer from methodological shortcomings by either using only cross-sectional data or focusing on longitudinal data from single countries thus limiting the generalizability of the findings. Therefore, it was the aim of this study to further investigate the association between psychiatric hospital bed numbers and prison population sizes based on a large longitudinal sample of 26 European countries over a timespan of almost 20 years.

## Methods

Data on psychiatric hospital beds and prison population were retrieved from Eurostat, the statistical office of the European Union (http://ec.europa.eu/eurostat/de). Data for these two main variables were available for the period between 1993 and 2011. Additionally, data on the following socioeconomic parameters were retrieved from Eurostat for the same time period: gross domestic product (GDP) per capita, unemployment rates, and the GINI coefficient as a measure of income inequality. Due to incomplete or breaks in time-series the following countries were excluded from further analysis: Belgium, Czech Republic, Hungary, Liechtenstein, Luxembourg, Macedonia, Norway, and Turkey. Finally, data from 26 European countries were included in the further analysis. Informed consent and institutional review board approval were not required because this was a retrospective analysis of publicly accessible data and individuals were deidentified by Eurostat, who provided the data to us. There were no human subjects concerns in this study.

### Analysis

Univariate associations between the dependent variable prison population and independent variables psychiatric beds, year, gross domestic product, GINI coefficient and unemployment rate were estimated by Spearman correlation coefficient for each country. The variables prison population and psychiatric beds were scaled by 100,000 inhabitants, gross domestic product (GDP) per inhabitant. The Wilcoxon signed rank test was used to test for a significant deviation of the mean of the correlation coefficients from zero.

The association between prison population and covariates was modelled by means of a fixed as well as a mixed random coefficient regression model in SAS (Software Version 9.4, 2002–2012 by SAS Institute Inc., Cary, NC, USA.) by procedure “mixed”. In this model, the variables gross domestic product, GINI coefficient, and unemployment rate were set as fixed, psychiatric beds and year were set as random coefficients with country as subject effect. The variance-covariance matrix of the fixed-effects parameter estimates were estimated by the empirical sandwich estimator. Only results of the mixed model are reported since the fixed as well as mixed model lead to very similar conclusions (data on request).

After eliminating non-significant variables from the model, the association between the number of psychiatric beds and prison population sizes are demonstrated by setting year to 2000 and plotting the estimated regression lines for each country. Finally, we show in a choropleth map the spatial distribution of the association of psychiatric beds and prison population sizes by plotting the estimated slopes (grouped into quartiles) for each country.

## Results

Descriptive statistics of the percentage changes of the number of psychiatric beds, sizes of prison population, GDP, GINI, and unemployment rate are shown in Table 1. The number of psychiatric beds decreased in all 26 countries ranging from -2.0% in Croatia to -88.2% in Italy. Changes in the sizes of prison population were more heterogeneous ranging from a considerable increase (+158.4% in Malta) to a marked decrease (-22.3% in Romania).

**Table 1:** Percentage changes of target variables for all 26 countries between 1993 and 2011.

Country	Psychiatric beds	Size of prison population	GDP	GINI	Unemployment rate
Austria	-34.2	+16.2	+42.7	+1.5	+7.7
Bulgaria	-31.9	+31.0	+76.2	+40.0	-31.1
Croatia	-2.0	+102.4	+62.3	+7.6	-13.3
Cyprus	-85.2	+141.0	+22.3	+0.7	+64.6
Denmark	-40.2	+11.6	+28.0	+39.0	+13.4
Estonia	-71.2	-14.4	+152.8	-11.4	-15.8
Finland	-50.6	-10.6	+38.5	+17.3	-49.4
France	-49.5	+18.7	+23.6	+6.2	-9.8
Germany	-12.6	+14.8	+28.2	±0	-29.3
Greece	-27.5	+68.5	+28.6	-4.3	+61.3
Iceland	-68.3	+67.4	+30.8	-2.1	+115.2
Ireland	-75.2	+0.6	+67.4	-9.7	+19.5
Italy	-88.2	+25.0	+14.1	-3.3	-25.0
Latvia	-50.4	-16.4	+36.2	+3.2	+14.9
Lithuania	-32.6	+11.7	+140.6	+6.5	+16.7
Malta	-19.4	+158.4	+13.4	9.3	-4.5
Netherlands	-21.6	+48.1	+40.7	11.0	-38.0
Poland	-11.8	+31.4	+97.6	+3.7	-11.0
Portugal	-37.7	+7.8	+22.5	7.6	+63.3
Romania	-18.4	-22.3	+64.3	+14.5	+18.0
Slovakia	-15.1	+33.4	+119.0	-1.9	+7.9
Slovenia	-18.9	+29.0	+58.8	+8.2	+18.8
Spain	-46.9	+28.3	+36.4	±0	+3.4
Sweden	-72.4	+8.8	+41.9	+16.2	-11.4
Switzerland	-22.7	-5.4	+16.2	-2.3	n.k.
United Kingdom	-43.6	+63.9	+46.4	+3.1	-20.6

Results of the Spearman correlation coefficients between psychiatric beds and salient variables based on country specific analysis are shown in Table 2. The mean and median of the Spearman correlation coefficients between psychiatric beds and prison population is -0.35 and -0.49 (p = <0.01), respectively, showing a significant negative association. Furthermore, prison population is significantly positively associated with year and GDP.

**Table 2:** Spearman correlations between prison population and other variables for all 26 countries.

with Variable	Min	Median	Mean	Max	SD	95% Lower CI	95% Upper CI	p-value
Psychiatric beds	-0.99	-0.49	-0.35	0.86	0.53	-0.56	-0.14	0.003
Year	-0.85	0.64	0.45	0.99	0.56	0.22	0.68	0.001
GDP	-0.94	0.55	0.35	0.90	0.58	0.11	0.58	0.011
GINI coefficient	-0.95	0.23	0.09	0.78	0.46	-0.10	0.27	0.325
Unemployment rate	-0.63	-0.06	-0.01	0.73	0.39	-0.18	0.15	0.744

**Fig 1:**
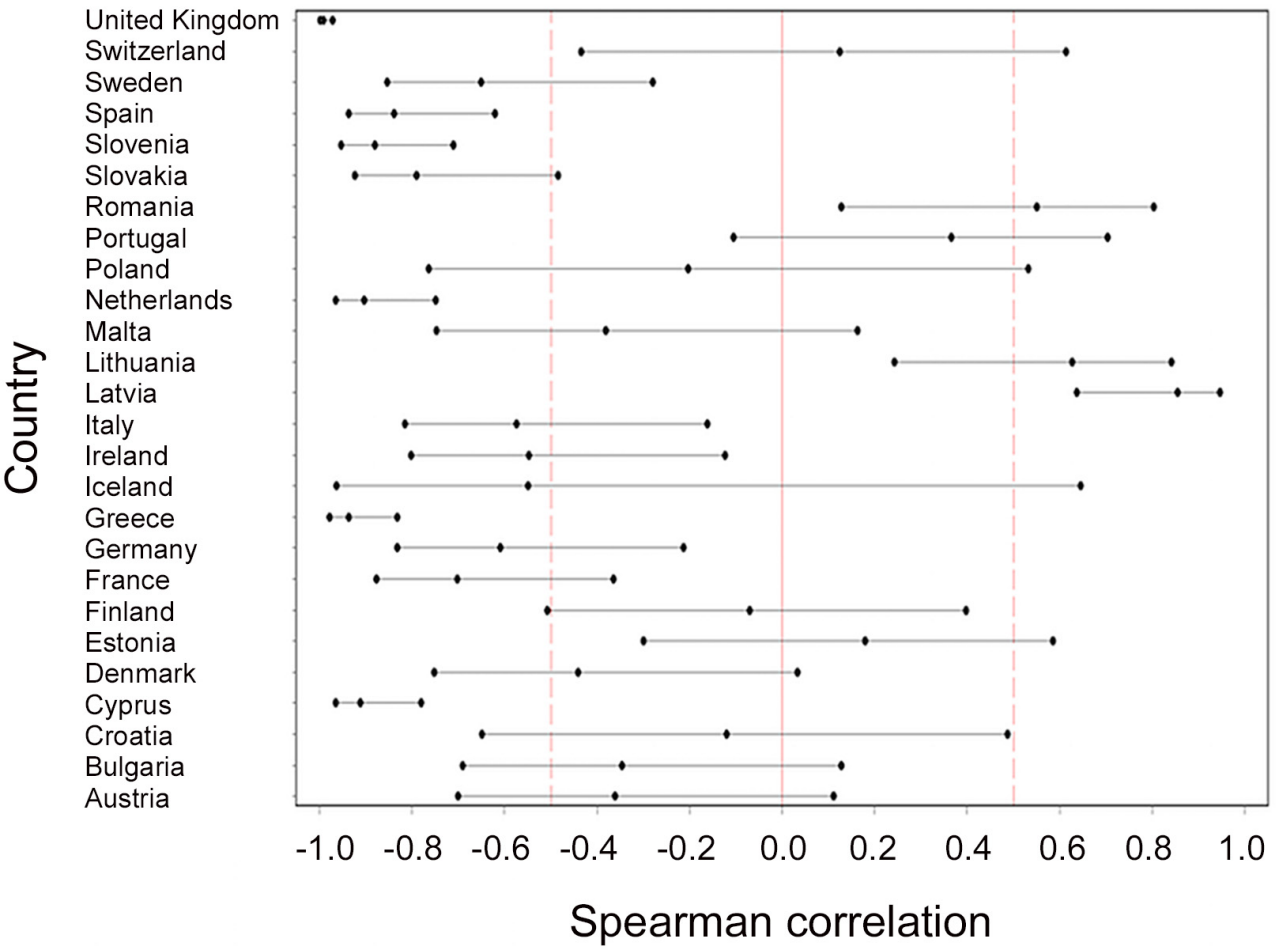
Spearman correlations between psychiatric hospital beds and prison population.

In Fig 1, the Spearman correlation coefficients of psychiatric beds with prison population along with its 95% confidence limits are shown for all 26 countries. Some countries exhibit a significant negative association whereas others show either not-significant or positive correlations.

In the mixed regression model with prison population as the dependent variable all the included variables showed not-significant fixed-parameter estimates: psychiatric beds: 0.04 (p = 0.88); year: 0.77 (p = 0.44); GDP: <-0.01 (p = 0.76); GINI coefficient: -0.35 (p = 0.82); unemployment rate: 0.43 (p = 0.57).

However, the covariance estimates for the random coefficients psychiatric beds (σ^2^ = 0.7488, p = <0.01) and year (σ^2^ = 0.0007, p = 0.03) were both significant at the 5% significance level.

**Fig 2:**
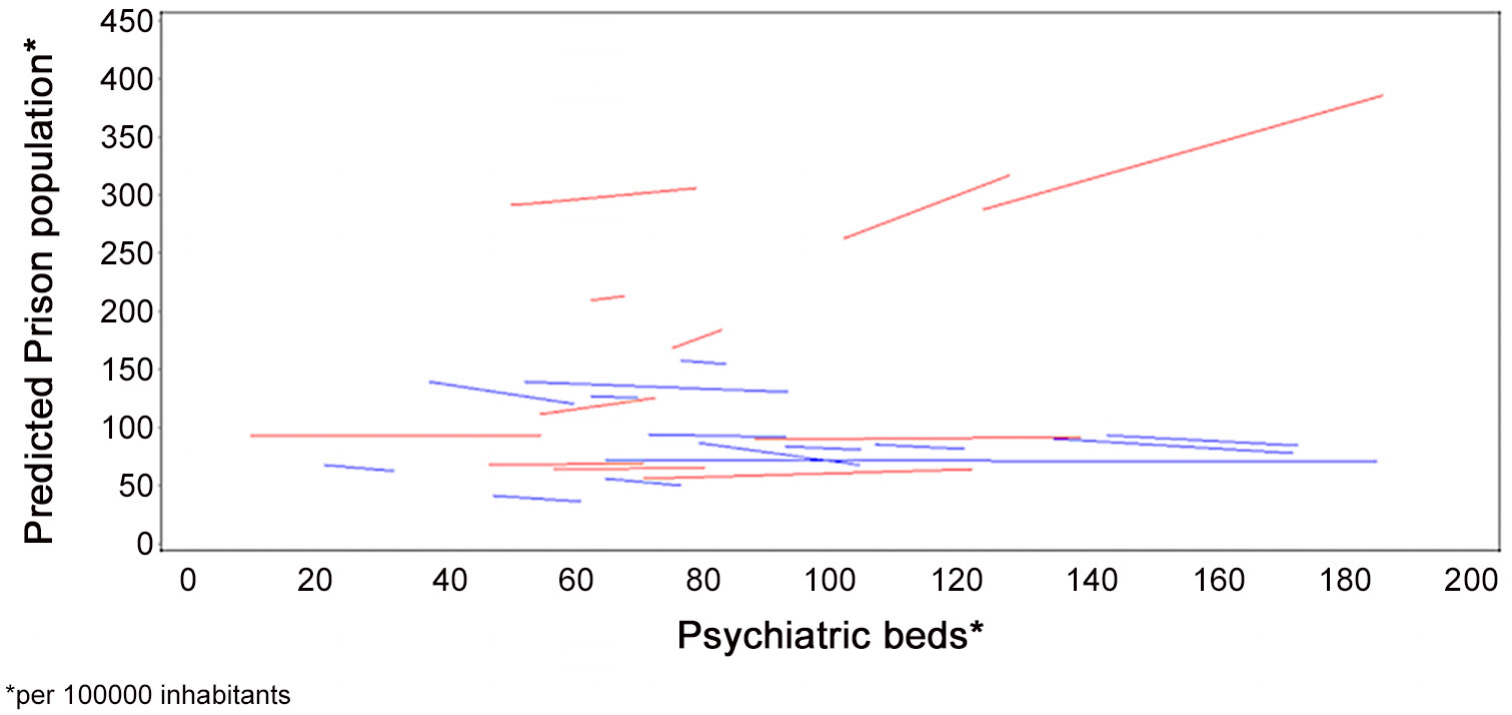
Estimated slopes for the effect of psychiatric beds on prison population with year set to 2000.

The estimated slopes for the effect of psychiatric beds on prison population with year set to 2000 are shown in Fig 2 for each country. Corresponding to the significant random coefficient for psychiatric beds, slopes are positive as well as negative showing clearly a heterogeneous effect of psychiatric beds on prison population.

**Fig 3:**
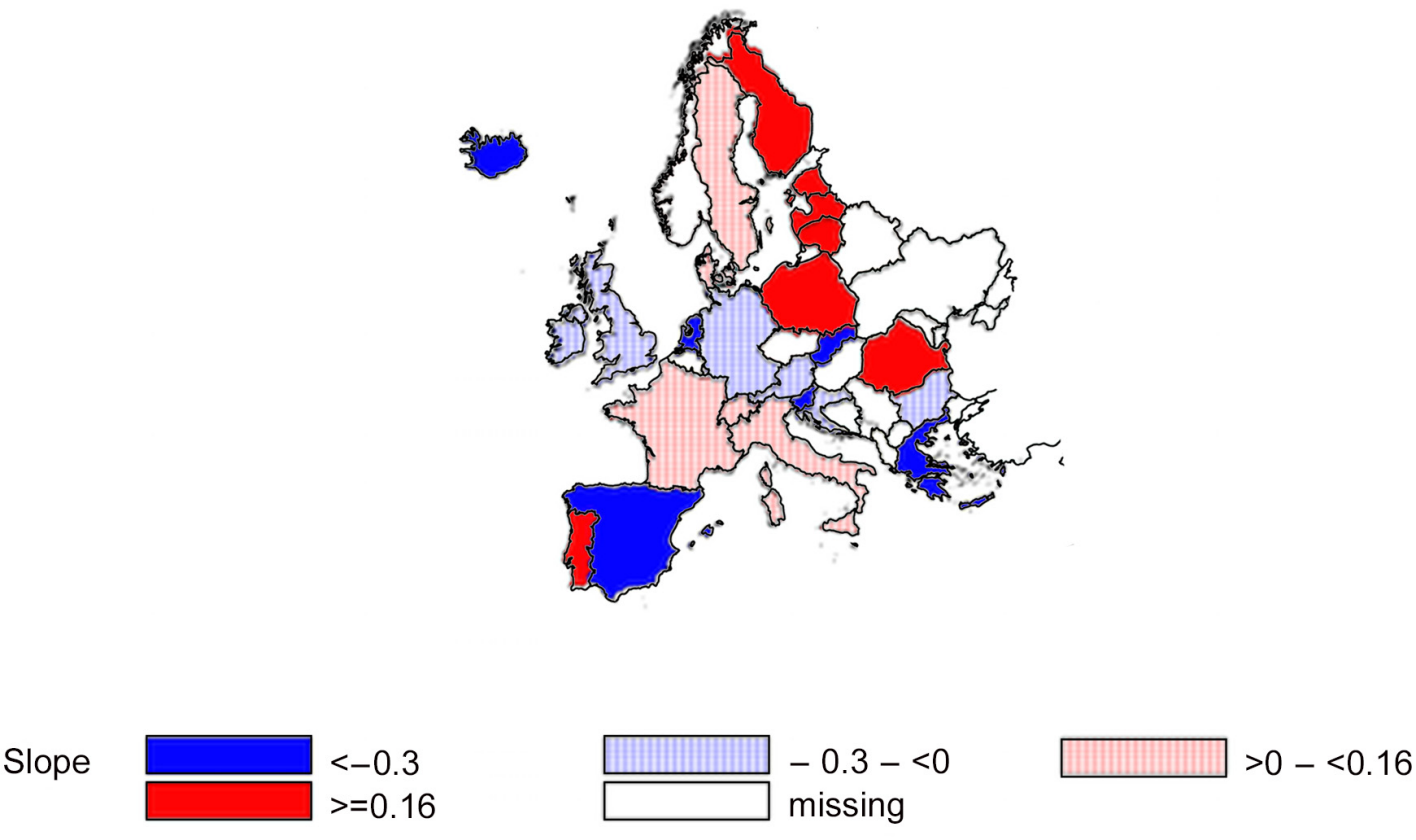
Spatial distribution of the slopes of the effect of psychiatric beds on prison population.

Finally, the spatial distribution of the slopes of the effect of psychiatric beds on prison population based on the mixed regression model is shown in Fig 3. Values below/above zero indicate a negative and positive association, respectively.

## Discussion

The aim of this study was to investigate the association between the number of psychiatric hospital beds and prison population sizes in 26 European countries over a time-period of almost two decades. To our knowledge this is the largest longitudinal investigation of the Penrose hypothesis to date. While Spearman correlation calculations showed a significant inverse relationship between the number of psychiatric hospital beds and the sizes of prison population, these associations failed to be statistically significant in a mixed multivariable regression model. We hereby show that a reduction of psychiatric bed numbers is not associated with an increased prison population per-se in our sample of European countries. Our findings point toward a distinctly more heterogeneous association between these two factors. Notably, the results of the random-effects model show a significant variation in the magnitude and direction of the association between countries. This variation cannot sufficiently be explained by socioeconomic indicators such as the GINI coefficient, GDP, or unemployment rates alone [18]. These results corroborate previous observations that the consequences of deinstitutionalization of the mental health system vary across countries likely due to specific features of their social welfare and health care system, national traditions, socio-cultural context, and the level of available resources [19]. Therefore, the notion of the Penrose hypothesis, which postulates a linear relationship between psychiatric hospital bed numbers and prison population sizes, seems to be an inadequate bivariate simplification of a rather complex and multifactorial relationship.

Our results are in contrast to the findings by Mundt et al. [12] who analyzed data of the sizes of prison population and the number of psychiatric beds of six South American countries and found a significant negative association between these two variables. Possible explanations for these diverging results include differences in the socioeconomic and political systems of the investigated countries. All six South American countries shared several important social and economic conditions and are classified by the UN as developing nations [12] [20]. In contrast, the sociopolitical development of the 26 European countries included in our study has been markedly diverse and heterogeneous over the last decades, most notably regarding the differences between former communist countries and Western European countries. Previous research already pointed to a more heterogeneous situation in post-communist countries with regards to the Penrose hypothesis [16]. Moreover, the process of deinstitutionalization of the mental health system in South America only gained momentum after 1990 [12]. In contrast, the process of deinstitutionalization of the mental health system in Europe already started in the 1960s. While the number of psychiatric hospital beds in the observed European countries further declined in the study period, it has been suggested that a process of re-institutionalization has been taking place in some European countries since the 1990s with rising rates of forensic beds and supervised and supported housing facilities for mentally ill patients [21] [22]. This also contrasts with the situation 75 years ago, when Penrose first postulated his hypothesis. At that time, big psychiatric asylums were the predominant agencies of psychiatric treatment and only very limited outpatient facilities or other mental health services were available.

However, the Penrose hypothesis suggests a certain constant number of individuals in society, both psychiatric patients and criminal offenders, in need of institutionalized care. Therefore, if a society does not provide adequate and well-resourced mental health care, individuals with norm-challenging behavior are in danger of being drawn into the criminal justice system [23]. As stated, many countries of the Western world have constantly reduced inpatient treatment time and the number of psychiatric beds during the last decades. Critical voices pointed out that these reforms overstate the original aims and especially patients suffering from the most severe form of psychiatric disorders are deprived of needed long-term inpatient treatment. The situation, known as “revolving door psychiatry” points to the need for increased allocation of resources for specific approaches for this psychiatric subpopulation [24]. Proper health care management in the community could reduce individual vulnerabilities to adverse circumstances that may lead to criminal offences.

On the other hand, the Penrose hypothesis, albeit unintentionally, in a certain sense equates criminal behavior and mental illness by depicting psychiatric beds and prison population as communicating vessels, thereby increasing the already existing stigma of mentally disordered patients as being violent and criminal [25, 26]. Hence, a careful interpretation of any inverse association of psychiatric beds and prison population is necessary as societal stigmatization increases barriers to help seeking behavior, leading to the fact that stigmatized patients avoid and withdraw from treatment [27]. In this sense, our results based on more comprehensive data, challenge the prevalent opinion that a reduction of psychiatric beds subsequently leads to increasing incarcerations.

### Strengths and limitations

There are several limitations to our study. Due to the ecological study design no causal relationships can be inferred from our results and the findings need to be interpreted with caution. Due to insufficient or inconsistent data 8 European countries had to be excluded from the analysis. All data were obtained from Eurostat, which in turn retrieves the data from national administrative agencies in the respective countries. Even though Eurostat uses quality assurance procedures, the quality of the retrieved data is difficult to assess due to the varying procedures by which the source data is gathered in the each country. Therefore, the comparability of the data between countries might be limited [16].

Furthermore, only linear associations between prison population and covariates have been estimated. For some countries this may not be optimal but necessary in light of the restricted numbers of parameters to be estimated. Finally, due to limited data availability, we did not include additional parameters for institutionalized mental health care besides number of psychiatric hospital beds such as forensic beds or supported housing facilities, which should be considered in future research.

Nevertheless, to our knowledge the sample size of 26 countries covering a timespan of almost 20 years makes our study the largest longitudinal investigation of the Penrose hypothesis to date. Additionally, the inclusion of important possible confounding factors such as GDP, GINI, and unemployment rate further strengthens our study.

## Conclusion

The Penrose hypothesis has been an important reference point for research into the complex relationship between the mental health system and the criminal justice system for more than 75 years [8]. While it has thus helped to generate more knowledge in this area of major socio-political significance, there is a considerable danger of oversimplification connected with it [28]. We especially caution against the possible unintentional stigmatization of mentally disordered individuals as criminals [29, 30]. Our results challenge the assumption of a direct link between psychiatric hospital bed numbers and the prison population sizes [17]. Further research including a more detailed analysis of political, legislative, and sociocultural aspects is needed in order to better understand this multifaceted relationship.
